# Nanoliposomal Encapsulation of *Capparis spinosa* Extract and Its Application in Jelly Formulation

**DOI:** 10.3390/molecules29122804

**Published:** 2024-06-12

**Authors:** Younes Zahedi, Rezvan Shaddel, Masoumeh Salamatian, Antoni Szumny

**Affiliations:** 1 Department of Food Science and Technology, Faculty of Agriculture and Natural Resources, University of Mohaghegh Ardabili, Ardabil P.O. Box 56199-11367, Iran; r.shaddel@uma.ac.ir (R.S.); m.salamatian1370@gmail.com (M.S.); 2 Department of Food Chemistry and Biocatalysis, Wrocław University of Environmental and Life Sciences, CK Norwida 25, 50-375 Wrocław, Poland

**Keywords:** *Capparis spinosa*, phenolic compounds, nanoliposome, jelly powder

## Abstract

This research aimed to encapsulate the *Capparis spinosa* fruit extract to increase its stability for incorporation into food products such as jelly or jelly powder. After extraction, the nanoliposomes containing the extract were prepared in ratios of 60-0, 50-10, 40-20, and 30-30 lecithin-to-cholesterol. The effects of lecithin-to-cholesterol concentrations on the related parameters were then evaluated. The results showed that the average particle size was in the range of 95.05 to 164.25 nm, and with an increasing cholesterol concentration, the particle size of the nanoliposomes increased. The addition of cholesterol increased the zeta potential from −60.40 to −68.55 millivolt. Furthermore, cholesterol led to an increase in encapsulation efficiency, and even improved the stability of phenolic compounds loaded in nanoliposomes during storage time. Fourier transform infrared (FTIR) spectroscopy confirmed the successful loading of the extract. Field emission scanning electron microscopy (FE-SEM) analysis revealed nano-sized spherical and almost-elliptical liposomes. For jelly powders, the water solubility index ranged from 39.5 to 43.7% (*p* > 0.05), and the hygroscopicity values ranged between 1.22 and 9.36 g/100 g (*p* < 0.05). In conclusion, nanoencapsulated *Capparis spinosa* extract displayed improved stability and can be used in jelly preparation without any challenge or unfavorable perception.

## 1. Introduction

The caper bush, scientifically known as *Capparis spinosa* L., is a plant of significant economic importance that belongs to the *Capparidaceae* family, which comprises over 250 species in the genus *Capparis*. The caper bush is a creeping shrub that grows to a height of 20 to 30 cm. The fruits of the caper bush are about 30 mm long and oval-shaped, with a greenish color and red pulp. The raw fruit of *C. spinosa* is high in fibers, crude oil, moisture, K, P, and Na, making it more suitable to pickle in the unripened stage. In addition, the pulp of the fruit is also rich in phenolic components such as quercetin and rutin. These phenolic components, along with carotenoids, tocopherols, and vitamin C, are recognized as factors that can contribute to the antioxidant activity of plant substances. Caper berries are traditionally used as a seasoning to garnish various dishes [1,2,3]. In addition to its culinary and nutritional uses, various folk therapeutic and medicinal properties have been attributed to the *C. spinosa* fruit. These properties include its potential to treat tuberculosis, atherosclerosis, hepatitis, kidney diseases, diabetes, hypertension, and cardiovascular complications, and to act as a diuretic. Capers have also shown potent antioxidant and anti-inflammatory features. These medicinal health functions and nutritional traits are mainly attributed to the presence of glucosides, alkaloids, essential fatty acids, reducing sugars, organic acids, terpenoids, vitamin C, resins, and flavonoids [1,2,3].

The application of natural polyphenols in foods or oral medications is limited due to their bitter taste, astringency, and lack of stability in the food matrix. Bioactive compounds like phenolics, flavonoids, carotenoids, vitamins, etc., are heat-labile components where the rate of deterioration varies with the process conditions applied. The loss of bioactive compounds during processing may be elevated or retarded by the product (such as jelly) composition like sugar, type and concentration of pectin, fruit and its cultivar, and pH. These changes may even continue during storage of the product, and again varying with the product composition, packaging material, and storage time and temperature. Furthermore, the biodisponibility of polyphenols in their unprotected form is typically low, mainly because of their low solubility in water. Bioactive compounds incorporated in the free form in foods can be excreted unmetabolized by humans. To overcome these drawbacks and increase polyphenol delivery and retention in cells and tissues, different delivery systems have been developed, and encapsulation would appear to be a promising route among them [4,5,6].

Encapsulation could affect bioavailability and bioaccessibility, as it guarantees the coating of the active substances and their controlled release, as well as their targeted deliverance to different areas of the digestive tract [7,8,9]. It is possible to use micro- and nanoencapsulation techniques. Nanocarriers produce more surface area compared with micron-sized carriers. As a result, they improve solubility, promote controlled release, increase bioavailability, and deeply facilitate the precision targeting of the encapsulated substances. Liposomes are a type of colloidal particles that consists of a membranous system composed of lipid bilayers that enclose one or more aqueous compartments, and nanoliposomes are the nanometric version of liposomes [10]. One of the advantages of liposomal delivery systems, compared with the other encapsulation techniques, is that they are made of substances that are beneficial for our health [10,11]. Nanoliposomes and liposomes have numerous potential applications in the food industry, including protecting unstable substances and improving the efficacy of food additives. The encapsulation of rutin to overcome the low water solubility and bioavailability of water [8], the encapsulation of betanin and its application in gummy candy [7], and the encapsulation of olive leaf phenolics for the enrichment of yogurt [5] are examples of food applications of nanoliposomes.

Jelly is a semi-solid product based on a hydrocolloid that establishes a network and generally consists of hydrocolloids as a gelling agent, edible acids, sweeteners, colorants, and flavorings agents. It is a preferred dessert among different age groups, owing mainly to its pleasant texture and digestibility. Some consumers take jelly products daily. However, consumers are constantly searching for healthier and tastier jellies with health-promoting attributes. Jelly desserts are conventionally made using edible gelatin, sugar, water, and flavorings [12]. Although jelly desserts are not typically considered to have a high nutritional value, their flexible formulation allows for the replacement of gel additives with natural antioxidants and vitamins from various sources, making them a potential candidate for producing healthy foods. In this manuscript, a pioneering approach to the utilization of *Capparis spinose* has been presented. Moving beyond the traditional use of the fruit in its pickled form, this research delves into the enhancement of jelly products through the incorporation of the fruit extract in a nanoliposomal encapsulated form. This novel technique not only augments the nutritional profile of the jellies but also imparts improved sensory qualities, such as enriched color and flavor, which are anticipated to elevate consumer appeal. The originality of this study is underscored by the application of nanotechnology for the encapsulation process, which has not been extensively explored in previous studies. The significance of our findings lies in the introduction of a fortified food product that marries the health benefits of *Capparis spinosa* with the cutting-edge science of nanoliposomes, offering a snack that is both nutritious and medicinally advantageous. This advancement represents a meaningful contribution to the field of functional foods and has the potential to positively influence dietary practices. Therefore, the main objectives of the present study were (1) to encapsulate *Capparis spinosa* extract in nanoliposomes to overcome both the instability of its bioactive compounds and the problems associated with polyphenols; (2) to develop a functional jelly product by embedding the nanoliposomes that have antioxidative activity and to evaluate the physicochemical properties of the jelly.

## 2. Results and Discussion

### 2.1. Extraction Efficiency, Phenolic Content, and Characterization of the Extract

The results of the ultrasound-assisted extraction process from *Capparis spinosa* fruit indicated that the maximum efficiency was 12.4% when the extraction time was 60 min. When the time was decreased to 30 min, the extraction efficiency dropped to 10.5%. In comparison, the efficiency of a simple extraction procedure involving only immersion in 60 °C distilled water for 30 and 60 min was measured at 5.1 and 6.6%, respectively, approximately half that of the ultrasound-assisted ones. This statement affirms that ultrasonic waves are more efficient than time alone in enhancing extraction efficiency. Additionally, ultrasonic waves serve as a driving force that facilitates the release of the fruit extract through the mechanisms outlined. The transmission of an ultrasonic wave through the solvent creates acoustic cavitation within the solvent, which is the primary mechanism for the enhanced extraction efficiency observed with ultrasound. Additionally, the contact surface area between the liquid and solid phases could be enhanced by ultrasound, which in turn promotes better solvent penetration into the sample matrix and facilitates the rapid diffusion of the solute from the solid phase to the solvent [13]. 

The ethanolic extract of the *C. spinosa* fruit yielded 6.3 mg of gallic acid equivalents (GAE) per gram of dry powder. In comparison, Bhoyar et al. [13] reported that the methanolic extract of the Indian *C. spinosa* fruit contained 4.7 mg GAE/g of dry sample. In another study, the total phenol content of the aqueous extract of the Algerian *C. spinosa* fruit was reported to be 7.2 mg of GAE/g dry sample [13]. Furthermore, the total phenol of the methanolic extract of the caper fruit ranged from 90 to 210 mg GAE/100 g of fresh weight for the Bahraini variety and 37 mg of GAE/100 g dry sample for the Turkish variety [2,14]. The different values obtained for the phenolic content may have various reasons; the total phenol content of *C. spinosa* may be influenced by the plant’s growth phase, different parts of the plant, and the geographical conditions. For example, the contents of carbohydrate and total phenol could be enhanced during fruit ripening [15]. Furthermore, there is a linear correlation between the phenolic content and antioxidant capacity, as approved in several studies. Tlili et al. [16] reported a linear correlation between the total phenolics of *C. spinosa* seeds and DPPH and ABTS assays. Arrar et al. [17] observed a correlation between the antioxidant activity and phenolic content of different parts of *C. spinosa*. Pearson’s correlation showed strong correlations between the total free phenolic of the Bahrain *C. spinosa* L. fruit and FRAP, DPPH, and ABTS assays [14]. Bhoyar et al. [13] found a high correlation between the DPPH, ABTS and FRAP assays and phenol as well as flavonoid contents. They concluded that the total phenolic contents can be used as indicators for antioxidant activities of edible parts of *C. spinosa*. The structures and chemical compositions of the active extract components could be closely affected by natural antioxidants. For this purpose, it is essential not only to measure the antioxidant activity of an extract based on its phenolic contents but also to characterize it thoroughly. It is important to note that other soluble compounds in various solvents, besides phenolic compounds, may also contribute to the high antioxidant capacity of an extract [2]. Conclusively, the obtained content of phenolic compounds in the extract of caper fruits falls in the range reported by others, making it potentially suitable for preparing functional foods. 

The berries of *C. spinosa* contain 2% proteins, 5% carbohydrates, 0.9% lipids, and 3% dietary fiber. They also have a moderate vitamin C content of 4 mg/100 g fresh weight [14]. Various sources in the literature indicate that fruit extract contains phenolic compounds such as quercetin and rutin, along with glucosides, alkaloids, essential fatty acids, reducing sugars, organic acids, terpenoids, resins, and flavonoids [18]. HPLC analysis of *C. spinosa* fruits revealed concentrations of 1019.52 ± 0.01 μg/g fresh weight for rutin and 97.86 ± 0.01 μg/g fresh weight for quercetin [19].

### 2.2. Particle Size, Particle Size Distribution, and Zeta Potential of Nanoliposomes 

Particle diameter and particle size distribution (PI) are two essential factors in determining the characteristics of colloidal systems, mainly depending on the preparation method and the particle composition [20]. The values of these parameters show the stability of colloidal systems and the encapsulation efficiency of polyphenols [21]. The results of the DLS measurements for the nanoliposomes produced are tabulated in Table 1. The Z-average value, indicating the average particle size of the particles, was in the range of 95.05 to 164.25 nm. As seen by improving the cholesterol concentration, the particle size of the nanoliposomes was enhanced. It can be stated that cholesterol can reduce the binding of hydrophobic molecules of the dual membrane through one of these mechanisms: (1) cholesterol competes with phospholipid molecules to capture the lipophilic space in the lipid membrane [22]; (2) cholesterol causes lipophilic molecules to attach to lipid membranes by reducing the flexibility of the bilayer membrane [23]. The increased size of nanoliposomes by the incorporation of cholesterol was also obtained by Malheiros et al. [24] for liposome-encapsulated nisin and Najaf-Najafi et al. [25] for the nanoliposomes loaded with Barije (*Ferula gummosa*) essential oil. 

Nanostructures in nature are commonly polydisperse. Measurement of the size distribution of the nanoparticle population is performed using the polydispersity index (P.I.). Theoretically, the P.I. < 0.5 indicates a suitable size distribution of a polydisperse system (P.I. > 0.5 indicates a broad size distribution). Considering this, the P.I. value for a monodisperse system is zero [26]. In the present study, the particle size distribution ranged from 0.3 to 0.5, which was within the acceptable range. The P.I. of the cholesterol-free nanoliposomes was maximal and was remarkably higher than those of the others. A significant decrease in the P.I. value of nanoliposomes was detected in the presence of cholesterol. With the addition of cholesterol to the nanoliposome membrane, although the particle diameter increased, it had a positive effect on P.I., reducing the size difference and smoothing the size of the nanoliposomes. The zeta potential is one of the factors that plays a critical role in the stability and electrostatic interactions of nanodelivery systems [21]. In general, particles are electrostatically stable if the zeta potential of the colloidal system is less than −30 mV and more than +30 mV [27]. The zeta potential value increased from −60.40 to −68.55 mV by adding cholesterol, indicating longer and longer-term stability of the particles. When the cholesterol ratio increased from 10 to higher levels, the enhancement of the potential zeta was statistically significant. Cholesterol stabilizes liposomes by tightening the membrane structure and increasing the zeta potential and electrostatic repulsion between particles [28]. In line with this study, Mohammadi et al. [29] reported that the zeta potential of empty lecithin-to-cholesterol nanoliposomes and vitamin-D3-loaded nanoliposomes moved from −30 to −45 mV with an increasing ratio of cholesterol in the membrane. In the current study, nanoliposomes created with only lecithin, which exhibited the smallest particle size, and those formulated with a 50-10 lecithin-to-cholesterol ratio, the smallest among those with cholesterol, were chosen for further analysis.

### 2.3. Encapsulation Efficiency (EE) and Loading Capacity (LC)

EE indicates the potential of wall materials to trap polyphenols, as well as the shelf life of the material enclosed in nanostructures [30]. The EE of nanoliposomes containing CSE with lecithin-to-cholesterol ratios of 60-0 and 50-10 was calculated as 89.3% and 95.7%, respectively. Since the hydrophilic space of the liposomes is greater than that of their hydrophobic space, the high percentage of encapsulation in both ratios can be due to the higher hydrophilic part of the extract and its connection to the inner and outer surfaces of the nanoliposomes, as well as its presence in the inner aqueous phase of the nanoliposomes. The presence of cholesterol in the wall material that led to the increase in efficiency may be due to the following: (1) the increased stability of the liposome membrane within the hydration stage, which could increase the EE because of the decreased permeability and increased membrane rigidity provided by cholesterol [31]; (2) the ability of cholesterol to cement the leakage space in the bilayer membranes, which could improve the EE and consequently enhance the level of extract in nanoliposomes [31]. 

Similarly, Alexander et al. [32] observed the positive effect of plant and animal sterols (cholesterol) on the EE of soy phospholipid nanoliposomes. Furthermore, according to the findings of Fan et al. [31], when the mass cholesterol-to-lipid ratio reached 0.25, the EE of salidroside-loaded nanoliposomes increased, but further increasing the cholesterol concentration led to disruption of the bilayer structure. 

Although phospholipids are endogenous and non-toxic biomaterials, the administration of lipids in the liposomal forms should not exceed the amounts that could be digested by the body. Therefore, it is advisable to keep the lipid load as low as possible in the formulations [33]. The LC of nanoliposomes containing CSE with lecithin-to-cholesterol ratios of 60-0 and 50-10 was calculated as 5.1% and 14.2%, respectively. The increase in the LC value of the nanoliposomes prepared with lecithin and cholesterol is related to their larger size. The LC and EE values could be affected by the lipophilic or hydrophilic characteristics of bioactive compounds and the critical factors of the liposome preparation process, such as surface area, aqueous volume, size, preparation method, and membrane rigidity [7]. In similar research, LC values for the phenolic components of green pistachio hull extract [34] and betanin [7] trapped in nanoliposomes of 40% and 26.8%, respectively, were obtained.

### 2.4. The Stability of Polyphenols during Storage

**Figure 1:**
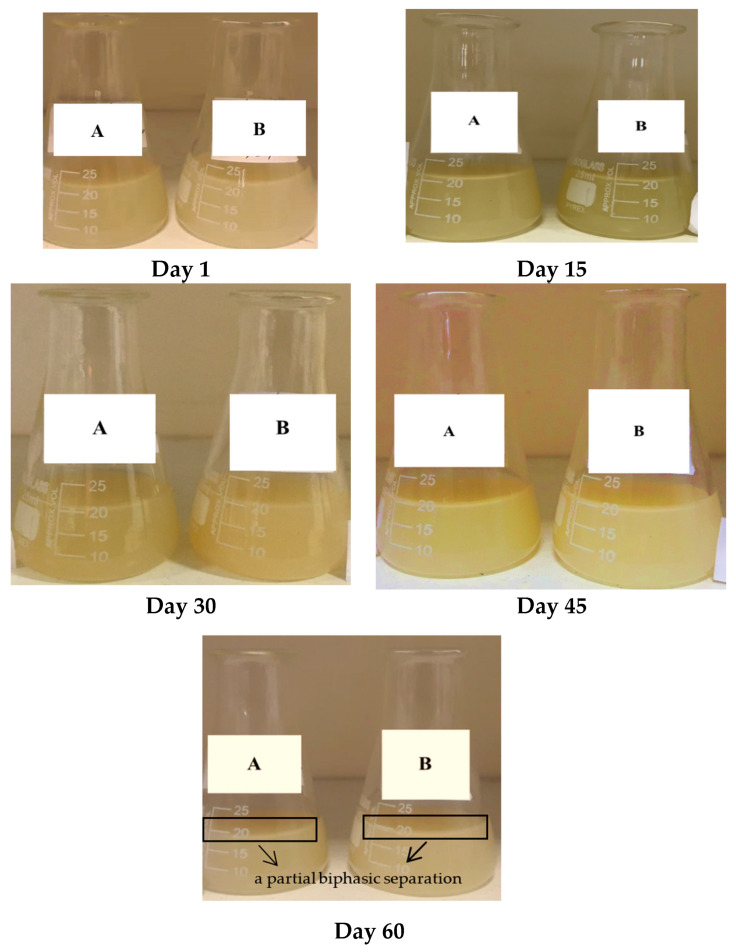
Physical stability of the solutions containing CSE-loaded nanoliposomes during storage for 60 days at room temperature; (A) nanoliposomes with a lecithin-to-cholesterol ratio of 60-0, (B) nanoliposomes with a lecithin-to-cholesterol ratio of 50-10.

Solutions containing *Capparis spinosa* extract-loaded nanoliposomes with lecithin-to-cholesterol ratios of 60-0 and 50-10 showed good physical stability; however, a partial biphasic separation was observed on day 60 of storage (Figure 1). The physical stability of the liposomes depends on the particle size, the number of layers, the structure of phospholipids, and the method of preparation [35]. 

**Figure 2:**
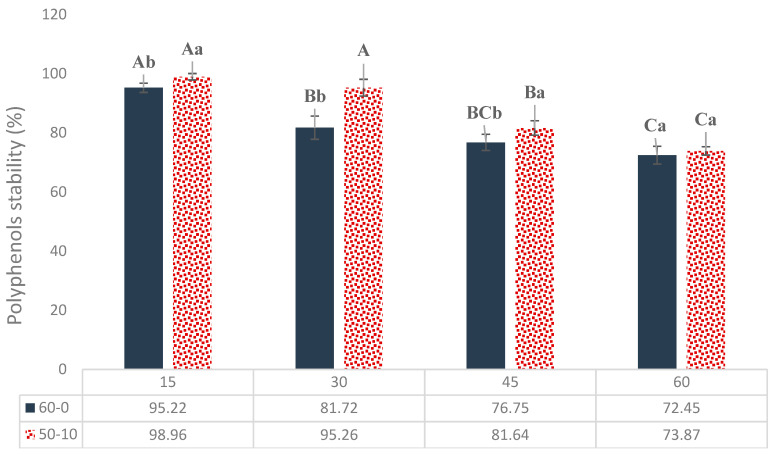
Stability of CSE-loaded nanoliposomes prepared in lecithin-to-cholesterol ratios of 60-0 and 50-10 during storage for 60 days at room temperature. Different lowercases and uppercases on the top of bars indicate significant differences (*p* < 0.05) within a day of storage (between treatments) and during storage, respectively.

The stability results of the phenolic compounds within 60 days (days 15, 30, 45, and 60) of storage at 25 °C revealed that until day 45 of storage, the stability of the phenolic components in the cholesterol-containing nanoliposomes (50-10) was significantly higher than the cholesterol-free nanoliposomes (60-0) (*p* < 0.05). However, no notable differences were observed in the stability percentage of phenolic compounds on day 60 of storage (Figure 2). Cholesterol affected the stability of the phenolic components loaded in nanoliposomes over storage time by decreasing the permeability and fluidity of the liposome membrane as well as by increasing the phospholipid phase transition temperature of the liposome; therefore, the entrapped materials cannot leak out easily. In addition, cholesterol also inhibits rupture and changes in the membrane. Furthermore, the last reason could be the increased zeta potential and electrostatic repulsion among phosphatidylcholine liposome membranes due to the presence of cholesterol [29]. 

The result of the statistical analysis showed that time was a significant factor in diminishing the stability of the phenolic compounds (*p* < 0.05). The stability of the phenolic compounds in both types of nanoliposomes decreased at an approximately similar rate during the storage period due to the oxidation of nanoliposomal phospholipids, which resulted in increased membrane permeability. The other reason for this could be the relatively high storage temperature of the samples (25 °C), leading to the gel-to-liquid transition of the lipid bilayers and consequently higher leakage [27]. Similarly to our research, Mohammadi et al. [29] found that the stability of vitamin-D3-loaded nanoliposomes with a lecithin-to-cholesterol ratio of 50-10 was higher than that of free cholesterol ones during 30 days of storage at refrigeration temperature. Only 5 to 9% of the stability was reduced due to the positive effect of cold temperature. Lu et al. [27] reported that the stability of nanoliposomes containing tea polyphenol after 30 and 60 days of storage at room temperature reached 88.2% and 78.5%, respectively. The stability was also 3–4 times higher at freezer temperature. In our research, the stability values were 95.2% (with cholesterol) and 81.7% (without cholesterol) on day 30, which decreased to 73.8% and 72.4% on day 60 of storage, respectively. Maintaining a lower temperature could improve stability. 

### 2.5. Fourier Transform Infrared Spectroscopy (FTIR)

**Figure 3:**
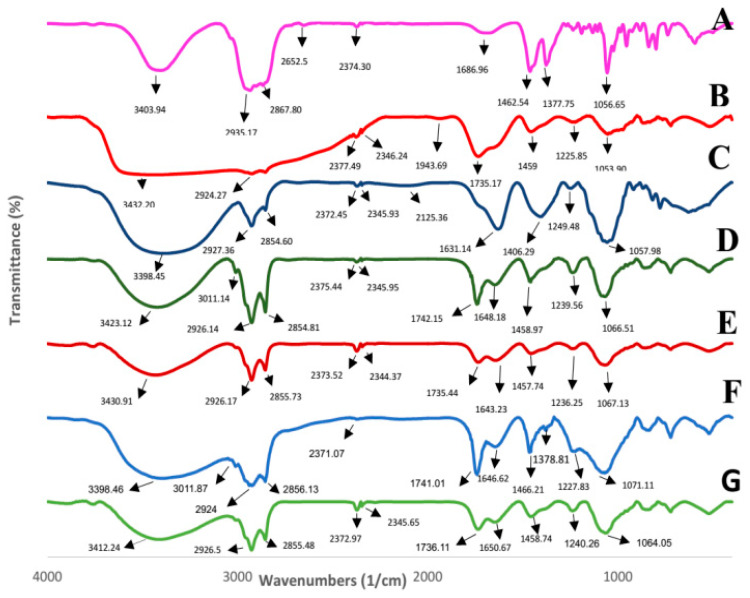
FTIR spectra for A = cholesterol, B = phosphatidylcholine, C = CSE, D = CSE-loaded nanoliposomes prepared in the lecithin-to-cholesterol ratio of 60-0, E = blank nanoliposomes prepared in the lecithin-to-cholesterol ratio of 60-0, F = CSE-loaded nanoliposomes prepared in the lecithin-to-cholesterol ratio of 50-10, and G = blank nanoliposomes prepared in the lecithin-to-cholesterol ratio of 50-10.

FTIR was performed to detect possible chemical interactions between the bioactive compounds and the carriers. The results of the FTIR analysis for CSE, wall materials, and unloaded or loaded nanoparticles are presented in Figure 3. The bands identified for cholesterol and phosphatidylcholine were almost close to those obtained by Gupta et al. [36] and Mohan et al. [37], respectively. The main cholesterol bands identified at 3403.94, 2935.17, 1685.96, 1462.54, 1377.75, 1191.61, 1056.65, and 839.17 cm^−1^ could be related to stretching HOH, asymmetric stretching vibrations of the CH2 and CH3 groups, C=C bond, symmetric stretching vibrations of CH3 and CH2, bending CH3 and CH2, C–C stretching, ring deformation, and C-C stretching, respectively. Also, the bands identified for phosphatidylcholine that were found at 3432.20, 2924.27, 1735.17, 1225.85, and 1057.98 cm^−1^ can be related to OH vibrations, asymmetric stretching vibrations of CH2, carbonyl group (C=O), and asymmetric PO2- and C-O in C-O-PO2-, respectively. According to reports by Nandiyanto et al. [38] and Fu et al. [39], the bands found at 3398.45, 1631.14, and 1057.98 cm^−1^ in the spectrum of CSE are indeed indicative of N-H stretching vibrations (amide I: NH_2_ group), C=C stretching vibrations (unsaturated bonds of aromatic rings), and C-O-C stretching vibrations (ether), respectively. FTIR analysis showed that by incorporation of the extract into the cholesterol-free nanoliposomes, a new peak was revealed at 3011.14 cm^−1^, and many of the absorption peaks were intensified. In the same way, two new peaks appeared at 1378.81 and 3011.87 cm^−1^, the absorption peak at 2345.65 cm^−1^ belonging to blank nanoliposomes disappeared, some absorption peaks were shifted, and many of them intensified in the spectra of nanoliposomes with a lecithin-to-cholesterol ratio of 50–10 (Figure 3). These changes are probably due to the interaction between the nanoliposome wall material and the CSE, confirming the successful loading of the extract. 

### 2.6. Field Emission Scanning Electron Microscopy (FE-SEM)

**Figure 4:**
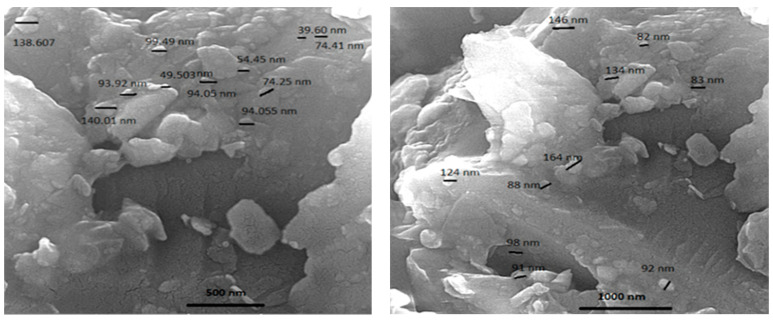
FE-SEM images of CSE-loaded nanoliposomes prepared in the lecithin-to-cholesterol ratio of 60-0.

FE-SEM analysis showed nanosized liposomes with acceptable dispersity. FE-SEM images (Figure 4) have been provided for the sample composed of a 60-0 lecithin-to-cholesterol ratio, which exhibits the smallest particle size among the nanoliposomes. The resulting morphology indicated that the particles are individually dispersed in the nanoliposomal system, and they are not agglomerated. The size of some liposomes measured in the images showed that most are less than 100 nm in diameter; however, several coarse particles were also found. Thus, the micrographs confirm the results provided using dynamic light scattering for the nanoliposome size (95 nm). As aforementioned, nanoliposome size is a crucial factor in demonstrating colloidal system stability and encapsulation efficiency. However, in the pharmaceutical field, it is a crucial factor in the efficient delivery of an active agent to the target. 

### 2.7. Physicochemical and Sensory Properties of the Jelly Samples

#### 2.7.1. Moisture Content (MC), pH, and Soluble Solid Content 

The results of the moisture content (MC), pH, and soluble solid content (Brix) measurements in different jelly samples are tabulated in Table 2. There was an insignificant difference between the MC of the control jelly and the jellies enriched with nanoencapsulated extract at both concentration levels (*p* > 0.05). It was expected that the MC of the treatments would be higher than the control due to the reduction of maltodextrin, while this happened only for the jellies containing the free extract. Samples containing free extract showed a significantly higher MC, probably due to their lower maltodextrin content, which binds fewer water molecules. In addition to this, the replacement of maltodextrin with the solution having less free extract plus more water increased the free water content of the F3 and F7 samples, which probably caused the shelf life of these jellies to decrease through the increase in a_w_, meaning that it is considered undesirable. The MC of the N3 and N7 jellies was low compared with the samples prepared with free extract, which may be due to the trapping of some water within the nanocapsules and also to the relative hydrophilicity of the wall materials. The pH of the jelly samples was in the range of 3.91–3.93, with insignificant differences (*p* > 0.05). This minor pH change was due to using the same amount of citric acid and fruit essence as pH-varying agents in the jelly formulations. In other studies, the range of pH variations for different jelly formulations, including low-calorie fruit jelly [40], sugar-free jelly [41], and citrus jelly [12], was between 3.19 and 3.67. The results of the Brix measurement revealed that there were no significant differences (*p* > 0.05) between different jelly samples, and the Brix values ranged from 31.25 to 33.25. The Brix degree of jelly is notably affected by recipe modification, and different values have been published for it in diverse jellies, including citrus jelly prepared with different sweeteners ranging from 20.1 to 23.1 [12], jelly containing pumpkin powder ranging from 14.5 to 15 [42], and low-calorie fruit jelly with different sweeteners ranging from 3.2 to 22.9 [40]. Conclusively, because the change in the pH and Brix may have adverse effects on product acceptance, the stability of these characteristics is considered a positive achievement.

#### 2.7.2. Color

The color of a jelly product may be the first and most important property for buying or eating. The results of the L*a*b* color parameters measurement for different jelly samples are tabulated in Table 2. As seen, no notable differences were observed in the L* values between jelly samples (*p* > 0.05), while a* and b* were significantly influenced by variations in formulation (*p* < 0.05). The addition of 3% *w*/*w* CSE in the formulation and the decrease in maltodextrin content caused a significant increase in a*, which did not show an effect of the encapsulation process at this level. With the enhancement of the extract concentration to 7% *w*/*w* and the decrease in maltodextrin to 1% *w*/*w*, the behavior of a* was changed entirely; the jelly loaded with nanoliposomes reached the maximum value of a*, while the non-encapsulated extract decreased the a* value of the jelly, and the final redness was the same as the control. This trend was observed for the b* value, i.e., the samples with a 7% *w*/*w* free extract indicated a decreased value compared with the others that contained the extract. Similarly to a*, with the replacement of some maltodextrin with CSE, the values of b* increased but were not significant, except for the F7 treatment. Mo et al. [43] reported that the a* and b* values of the jellies containing 0.5 to 3% *v*/*v* of sedum extract were significantly higher than those of the control. In general, a noticeable change was not detected in the jellies’ color after the addition of free or encapsulated CSE at two concentrations, and among four different jelly formulations, the F7 color was as most similar to the control. 

#### 2.7.3. Texture Profile Analysis (TPA)

The mean values of the parameters of the textural profile (hardness, springiness, and cohesiveness) are presented in Table 2. It was expected that the texture characteristics of the jellies would undergo some changes in response to the reduction of maltodextrin as a moisture absorbent, but such phenomena were not detected. Both the content and the form of the application of CSE in jelly formulations did not create a notable change in the three measured parameters (*p* > 0.05). Regarding the importance of texture in the acceptance of jelly, every variation in texture could be challenging if the samples were influenced by the extract. The use of instrumental techniques for evaluating sensory properties is usually preferable to sensorial evaluations due to the poor reproducibility, time-consuming nature, and high costs of sensorial evaluations [44]. In this study, instrumental texture results were well confirmed by sensorial texture values. Consistent with this study, the incorporation of encapsulated anthocyanin into jellies prepared with different ratios of gelatin, gum Arabic, and maltodextrin did not significantly change the textural traits. The values of hardness, springiness, and cohesiveness were in the range of 15.7–15.83 N, 0.045–0.058%, and 1.03–1.23, respectively [45]. Overall, considering that the control jelly is prepared based on a standard formula, if the textural parameters do not change as a result of the extract addition, it will be a favorable result.

#### 2.7.4. Syneresis

Syneresis is a significant process that occurs in aqueous physical gels, and it is closely associated with flavor release and other food-related applications. During syneresis, liquid is expelled from many solid products, such as jellies, jams, and dairy products [46]. In this study, no syneresis was detected for different jelly formulations, indicating that various levels of maltodextrin could not affect syneresis, and gelatin was the prime factor in controlling it, which is considered a desirable result. Gelatin gels that have a pH that is lower or higher than the isoelectric point tend to swell significantly. However, they are stable and do not exhibit syneresis, even at low protein concentrations [46]. Furthermore, different jellifying agents show completely different behavior within the jelly matrix; for example, Kim et al. [47] stated that the syneresis values of the jellies prepared with gelatin (0.27–0.83%) were lower than those of those prepared with konjac and carrageenan gums.

#### 2.7.5. Sensorial Evaluation

Sensorial evaluation suggests the opportunity to collect complete analyses of the different features of jelly as perceived by the human sense. Table 3 shows the mean scores for the sensorial evaluation parameters. The panelists did not judge significant distinctions observed in color and texture properties between samples (*p* > 0.05). However, the flavor of the N3 sample was significantly lower than that of the F3 and control samples (*p* < 0.05). By improving the extract concentration in the jelly formulation, the bitter taste of the phenolic compounds was achieved to some extent in the F7 sample. However, it received good scores from the panelists. All jellies were rated favorably for their general acceptability, and there were no significant differences in the mean scores between them based on statistical analysis. Overall, different jelly samples had the same acceptance, as was reflected by the panelists’ liking scores, demonstrating that neither the content nor the application form of the CSE negatively impacted the sensory characteristics, and by reducing maltodextrin and adding bioactive compounds, functional jellies with satisfactory sensory properties can be produced.

### 2.8. Jelly Powders Attributes

#### 2.8.1. Water Solubility Index (WSI)

A high WSI is desirable because it indicates that the powder can be easily dissolved in water and other aqueous solutions, leading to quick and complete reconstitution [9]. The WSI of different jelly powders is shown in Table 4. As seen, there were no significant differences among the WSIs of different formulations (*p* > 0.05), and the WSI values ranged from 39.5 to 43.7%. In similar research, the WSI of spray-dried jelly powders containing encapsulated barberry’s anthocyanin and spray-dried pomegranate juice powders ranged from 94.8 to 96%, and more than 90%, respectively [9,45]. It seemed that decreasing the carbohydrate content and incorporating CSE, especially in the form of a nanocapsule, would have a negative effect on the WSI of the jelly powders, while, overall, the WSI of all the powders was satisfactory.

#### 2.8.2. Hygroscopicity of Jelly Powders

Powder products provided by spray-drying may have several problems, such as solubility, hygroscopicity, and stickiness, due to the presence of acids and sugars, which have low glass transition temperatures [48]. The hygroscopic behavior of different jelly powders is presented in Table 4. Hygroscopicity values ranged between 1.22 and 9.36 g/100 g, and the control sample significantly absorbed more moisture than the others (*p* < 0.05). The reason for the high hygroscopicity of the control sample was probably a higher concentration of maltodextrin. When 3% maltodextrin was replaced with CSE, a significant decrease was detected in the hygroscopic potential of jelly powders. Although replacement of the carbohydrate used with the extract resulted in a further decrease in the powder’s hygroscopicity, the differences between powder samples containing different contents of CSE were not significant (*p* > 0.05). Also, using the extract as free or encapsulated in the jelly formulation was inefficient in changing the hygroscopic properties. We must take into account that the form of the application of maltodextrin can influence its hygroscopic behavior, and many studies have shown that maltodextrin is an efficient carrier agent for spray-dried powders due to its low hygroscopicity [48]. For example, Cai and Corke [49] stated that coating spray-dried betacyanin powders with 15 DE maltodextrin caused a reduction in the powder’s tendency to moisten. Furthermore, maltodextrin, when used as a wall material for barberry anthocyanins, did not increase the hygroscopicity of the spray-dried pigment powder [45]. Higher moisture absorption of a powder can promote the degradation rate; thus, an extended shelf life is obtained concerning the reduced hygroscopicity of the extract-loaded jelly powders. 

## 3. Materials and Methods

### 3.1. Materials

All chemicals and reagents used were of analytical grade. Ethanol (96%), chloroform, and glacial acetic acid were purchased from Dr. Mojallali company (Tehran, Iran), sodium carbonate was purchased from Arvin Chemical company (Tehran, Iran), sodium benzoate, gallic acid, Tween 80, and Folin–Ciocalteu were purchased from Merck KGaA (Darmstadt, Germany), cholesterol and L-α-phosphatidylcholine (soybean) were purchased from Sigma-Aldrich Chemie Gmbh (Schnelldorf, Germany), and the Amicon Ultra-4 centrifugal filter device and polyvinylidene fluoride (PVDF) syringe filter were purchased from Millipore (Millipore, Bedford, MA, USA). The *Capparis spinosa* fruits were harvested from Ilam province (Iran) in summer and kept at −20 °C until use.

### 3.2. Extract Preparation

*The Capparis spinosa* fruits were dried with a freeze-dryer (Christ, Alpha 1–4 LD, Martin Christ, Germany) and ground using a grinder. Then, 1000 mL ethanol of was added to 5 g of fruit powder (20:1), and ultrasound-assisted extraction was carried out using an ultrasonic cleaning bath (Backer, vCLEAN 1 L6, Tehran, Iran) with 40 kHz at 30 °C for 30 and 60 min. The extracted solution was filtered using filter paper and lyophilized by a laboratory lyophilizer (Zirbus, Vaco 5, GmbH, Bad Grund/Harz, Germany).

### 3.3. Phenolic Content Measurement

The Folin–Ciocalteu method was used to determine the phenolic content of the extract [50]. To generate a standard curve, gallic acid (0–200 ppm) was applied. 

### 3.4. Preparation of Nanoliposomes Containing Capparis spinosa Fruit Extract

Nanoliposomes comprising *Capparis spinosa* fruit extract (CSE) were manufactured in varying ratios of 60-0, 50-10, 40-20, and 30-30 (mg *w*/*w*) of lecithin-to-cholesterol. For this purpose, after preparing a 250 mM sodium acetate buffer and adjusting its pH to 3.5 (827 pH meter, Metrohm Ltd, Herisa, Switzerland), different amounts of lecithin in the buffer were mixed on a hot plate magnetic stirrer (Alpha, D500, Tehran, Iran) at 45 °C for 2 h and placed in the refrigerator overnight to be well hydrated. Then, 25 mg of extract, 20 mg Tween 80, and the mentioned cholesterol ratios were added to 10 mL of buffer containing the hydrated lecithin with different ratios. The resulting blend was stirred with the hot plate magnetic stirrer at 35 °C. Upon complete mixing, the particle size was reduced to the nanoscale with an IKA T25 digital homogenizer (Ultra-Turrax, Jahnke und Kunkel GmbH, Staufen, Germany) for 5 min at 20,000 rpm at a temperature exceeding the phase transition temperature of lecithin. The final step was the ultrasonic process for 5 min at a frequency of 40 kHz in cycles of 15 s on and 5 s off, after which nanoliposomes were obtained [51]. 

### 3.5. Evaluation of Nanoliposomes Containing Capparis spinosa Extract

#### 3.5.1. Particle Size, Particle Size Distribution, and Zeta Potential

The particle size, particle size distribution, and zeta potential of the liposomes were determined using a dynamic light scattering (DLS) device. To prevent the nanoparticles from clumping together and to ensure that they are adequately separated, the samples containing nanoliposomes were thinned out using a tenfold volume of distilled water. Following dilution, these samples were introduced into a capillary tube for examination with a DLS device (Horiba, SZ-100, Kyoto, Japan) [52].

#### 3.5.2. Encapsulation Efficiency (EE) of Polyphenols and Loading Capacity (LC) of Nanoliposomes

The encapsulation efficiency (EE) of polyphenols was determined using the method described by Tavakoli et al. [5]. Initially, the free (unencapsulated) phenolic compounds were separated from the entrapped parts by placing a certain amount of CSEs into an Amicon Ultra-4 centrifugal filter device with a cut-off of 10 kDa, followed by centrifugation (Hettich Universal, vs. 15,000 CFN II, Randreas Hettich GmbH & Co., Tuttlingen, Germany) at 5590 rcf for 15 min. Subsequently, 0.1 N chloroform was added to the encapsulated phase to dissolve the liposomal membrane and release the phenolic compounds. Finally, the solution was filtered using a 0.45 μm polyvinylidene fluoride (PVDF) syringe filter. The concentration of phenolic compounds in each sample was determined based on the gallic acid standard curve, as mentioned in Section 3.3. The EE was measured using the following Equation (1):

(1) $$\mathrm{EE}\;\left(\%\right)=\frac{C_{\mathrm{total}\;}-{\;C}_{\mathrm{free}}}{C_{\mathrm{total}\;}}\times 100$$

where C_total_ is the concentration of the total phenol content in the liposomal suspension, and C_free_ is the concentration of the free phenolic content. 

The LC of nanoliposomes was estimated by the following Equation (2):

(2) $$\mathrm{LC}\;\left(\%\right)=\frac{\mathrm{Content}\;\mathrm{of}\;\mathrm{the}\;\mathrm{extract}\;\mathrm{entrapped}\;\mathrm{in}\;\mathrm{liposomes}}{\mathrm{Total}\;\mathrm{lipid}\;\mathrm{content}}\times 100$$

#### 3.5.3. Stability of Polyphenols during Storage

To assess the physical stability of the samples at room temperature within 60 days, visual observation of phase separation (precipitation formation check) was used. Furthermore, the chemical stability of the phenolic compounds was monitored by studying their leakage from the nanoliposomes using a spectrophotometer (Bel Photonics, UV-M51, Monza, Italy) at 765 nm within 60 days (days 15, 30, 45, and 60) of storage at 25 °C, through the following Equation (3):

(3) $$\mathrm{Stability}\;\left(\%\right)=\frac{\mathrm{Remained}\;\mathrm{phenolics}}{\mathrm{Initial}\;\mathrm{encapsulated}\;\mathrm{phenolics}}\times 100$$

#### 3.5.4. Fourier Transform Infrared (FTIR) Spectroscopy

FTIR spectroscopy (RXI, Perkin Elmer, Naperville, IL, USA) was performed at a frequency of 400–4000 cm^−1^ for each material, including cholesterol, phosphatidylcholine, freeze-dried samples of CSE-loaded nanoliposomes, and blank nanoliposomes. 

#### 3.5.5. Field Emission Scanning Electron Microscopy (FE-SEM)

The morphology of CSE-loaded nanoliposomes prepared in a lecithin-to-cholesterol ratio of 60-0 was observed using an FE-SEM instrument (MIRA3 FEG-SEM, Tescan Co., Brno, Czech Republic). For this purpose, a laboratory slide was used to deposit a drop of nanoliposomal solution, which was then allowed to dry at room temperature. Subsequently, an ion-sputtering apparatus was used to deposit a layer of gold onto the sample for viewing under electron microscopy. The scan was performed at magnifications of 30 and 50 K×.

### 3.6. Production of Jelly Containing Capparis spinosa Fruit Extract

Jelly samples were produced according to the Iran Standard Institute (No. 2689) [53]. Table 5 contains the formulations for five different types of jelly treatments. CSE was applied as two forms of encapsulated and free at two concentrations of 3 and 7% *w*/*w* of jelly. The nanoliposome with a lecithin-to-cholesterol ratio of 50-10 was selected for loading of the extract and preparation of jelly samples. The pH of the jelly solutions was adjusted to 4.3 using citric acid solution (20% *w*/*v*), and sodium benzoate (0.05% *w*/*v*) was applied as a preservative. 

### 3.7. Jelly Evaluations

#### 3.7.1. Moisture Content

The moisture content of the jelly samples was determined by an oven-drying method at 103 ± 2 °C. 

#### 3.7.2. pH

pH measurements were made with a probe pH meter (Testo 230; Testo AG, Lenzkirch, Germany).

#### 3.7.3. Soluble Solid Content

The solid soluble content of the jelly formulations (before setting) was measured at room temperature using a refractometer (Huixia, SBR-90A, Fuzhou, China). 

#### 3.7.4. Color 

The jellies’ color (L*a*b* values) was measured by a CR-400 chroma meter (Minolta, Kyoto, Japan) on the cubic samples (20 × 20 × 20 mm^3^) with three replications. The L* value is the lightness component, which ranges from 0 to 100, and the parameters a* (green to red or redness) and b* (blue to yellow or yellowness) are the two chromatic components, which range from −120 to +120.

#### 3.7.5. Texture Profile Analysis (TPA)

The texture properties were assessed using a texture analyzer (Santam Design & Manufacturing CO, STM-20, Tehran, Iran) equipped with a 25 kg load cell according to the method described by Periche et al. [54]. 

#### 3.7.6. Jelly Syneresis

To assess the syneresis in jelly samples, two distinct methodologies outlined by Akesowan [55] and Khouryieh et al. [41] were employed. Syneresis of the samples was calculated as the following Equation (4) after three replications:

(4) $$\mathrm{Syneresis}\;\left(\%\right)=\frac{\mathrm{Total}\;\mathrm{weight}\;\mathrm{of}\;\mathrm{separated}\;\mathrm{liquid}\;\left(g\right)}{\mathrm{Total}\;\mathrm{weight}\;\mathrm{of}\;\mathrm{jelly}\;\left(g\right)}\times 100$$

#### 3.7.7. Sensorial Evaluation

A taste panel was held with ten panelists, including five women and five men between the ages of 25 and 30. The panelists were chosen from among staff and students at the University of Mohaghegh Ardabili. The panelists used a 5-point hedonic scale sensory test to rate the samples, with 5 indicating “like very much”, 4 indicating “like moderately”, 3 indicating “neither like nor dislike”, 2 indicating “dislike moderately”, and 1 indicating “dislike very much”. Average scores obtained from the sensory evaluation were used in the analysis.

### 3.8. Powder Production

The different jelly formulations were dried by a freeze-dryer (Christ, Alpha 1–4 LD, Martin Christ, Germany). The quality of the powder samples obtained was evaluated by two tests (water solubility index and hygroscopicity) as follows: 

#### 3.8.1. WSI (Water Solubility Index)

The water solubility index (WSI) of the jelly powders was determined using the method described by Akhavan-Mahdavi et al. [45]. First, 2.5 g of the powder was mixed thoroughly by a vortex for 1 min in 30 mL of distilled water in a 50 mL centrifuge tube. The centrifuge tube was then incubated at 37 °C in a water bath for 30 min. Next, the solution was centrifuged (Armaghan Teb Iranian, Tehran, Iran) at 12,297× *g* for 20 min. Finally, the supernatant was collected and vacuum-dried in an oven at 105 °C. The WSI was calculated using the following Equation (5):

(5) $$\mathrm{WSI}\;\left(\%\right)=\frac{\mathrm{Dried}\;\mathrm{supernatant}\;\left(g\right)}{\mathrm{Jelly}\;\mathrm{powder}\;\left(g\right)}\times 100$$

#### 3.8.2. Hygroscopicity

An amount of 2 g of the jelly powder was placed in a chamber containing a saturated solution of NaCl at 25 °C and weighed until equilibrium was reached. Hygroscopicity was expressed as grams of adsorbed moisture per 100 g of dry solids (g/100 g) [45]. 

### 3.9. Statistical Analysis

The collected data were analyzed using a one-way analysis of variance (ANOVA) with a completely randomized design (CRD). A repeated measures two-way ANOVA procedure was used to statistically analyze the obtained data for the “stability of polyphenols during storage” experiment. The means were compared using Duncan’s test, with statistical significance set at *p* < 0.05. Statistical analysis was performed using version 9.1.3 of the SAS statistical program. 

## 4. Conclusions

In the present study, lecithin-to-cholesterol nanoliposomes were successfully loaded with an ethanolic extract of the *Capparis spinosa* fruit in the acceptable particle size. Complementary tests also confirmed the success of CSE nanoencapsulation. In spite of achieving the same results for most of the physicochemical properties of the jelly samples loaded with free or nanoencapsulated CSE, we suggest the use of the encapsulated form because of well-protected phenolic components during storage. Therefore, despite the increase in the cost of jelly production, functional food developers can safely claim that this new product is relatively low-calorie, profitable, and nutraceutical. 

Some people may not prefer the consumption of pickled or salted *Caper* berries with food, so by introducing a part of its functional ingredients in the jelly, they will also benefit from them. Furthermore, a new market will be created for *Capparis spinosa* fruit, which grows wild in some countries. 

The extract-loaded jelly powders indicated a decreased hygroscopicity, which could be interpreted as a positive effect. Overall, encapsulating the phenolic compounds can improve their stability and widen their commercial application. It is suggested to use an electronic tongue to better understand the bitterness or astringency of CSE-loaded nanoliposomes in future studies.

## Figures and Tables

**Table 1:** Average particle size, particle size distribution, and zeta potential of nanoliposomes prepared with different ratios of lecithin-to-cholesterol loaded with CSE *.

Lecithin: Cholesterol (*w*:*w*)	Z-Average (nm)	Polydispersity Index (P.I)	Zeta Potential (mv)
60:0	95.05 ± 5.3 ^c^	0.517 ± 0.06 ^a^	−60.40 ± 0.4 ^b^
50:10	118.5 ± 0.3 ^b^	0.329 ± 0.02 ^b^	−63.05 ± 2.7 ^b^
40:20	130.05 ± 5.6 ^b^	0.382 ± 0.02 ^b^	−68.55 ± 0.3 ^a^
30:30	164.25 ± 14.5 ^a^	0.364 ± 0.05 ^b^	−68.45 ± 1.2 ^a^

* Different small letters in each column indicate significant differences at a confidence level of 5%.

**Table 2:** Physicochemical property measurement results of different jelly samples with or without CSE *.

Sample **	Moisture Content (%)	pH	Brix (^o^)	L* (Lightness)	a* (Redness)	b* (Yellowness)	Hardness (N)	Cohesiveness	Springiness (%)
Control	7.95 ± 0.1 ^b^	3.93 ± 0.001 ^a^	33.25 ± 0.1 ^a^	44.99 ± 2.1 ^a^	0.01 ± 0.1 ^c^	13.68 ± 0.2 ^ab^	21.47 ± 0.4 ^a^	0.48 ± 0.08 ^a^	1.01 ± 0.03 ^a^
N3	6.68 ± 0.9 ^b^	3.92 ± 0.005 ^a^	32.00 ± 0.2 ^a^	43.04 ± 3.6 ^a^	0.39 ± 0.2 ^b^	14.65 ± 1.5 ^a^	24.59 ± 0.6 ^a^	0.52 ± 0.06 ^a^	1.00 ± 0.01 ^a^
F3	12.65 ± 0.1 ^a^	3.91 ± 0.005 ^a^	32.75 ± 0.2 ^a^	45.62 ± 0.9 ^a^	0.37 ± 0.0 ^b^	14.92 ± 0.6 ^a^	26.90 ± 0.9 ^a^	0.46 ± 0.14 ^a^	0.91 ± 0.18 ^a^
N7	7.12 ± 0.2 ^b^	3.91 ± 0.010 ^a^	31.25 ± 0.0 ^a^	42.57 ± 0.7 ^a^	0.85 ± 0.1 ^a^	14.78 ± 0.3 ^a^	22.30 ± 0.9 ^a^	0.47 ± 0.08 ^a^	0.99 ± 0.01 ^a^
F7	10.95 ± 0.2 ^a^	3.91 ± 0.002 ^a^	32.00 ± 0.1 ^a^	46.11 ± 3.4 ^a^	0.05 ± 0.1 ^c^	12.64 ± 0.3 ^b^	24.91 ± 0.6 ^a^	0.42 ± 0.13 ^a^	1.00 ± 0.02 ^a^

* Different small letters in each column indicate a significant difference at the confidence level of 5%. ** N3: jelly containing 3% *w*/*w* nanoencapsulated CSE, F3: jelly containing 3% *w*/*w* free CSE, N7: jelly containing 7% *w*/*w* nanoencapsulated CSE, F7: jelly containing 7% *w*/*w* free CSE.

**Table 3:** Evaluation results of sensory characteristics of different jelly samples with or without CSE *.

Sample **	Color	Flavor	Texture	Overall Acceptability
Control	3.87 ± 0.9 ^a^	3.87 ± 0.8 ^a^	3.62 ± 0.5 ^a^	3.75 ± 1.0 ^a^
N3	3.75 ± 0.9 ^a^	2.75 ± 1.0 ^b^	3.62 ± 0.7 ^a^	3.75 ± 0.7 ^a^
F3	4.12 ± 0.6 ^a^	4.12 ± 0.6 ^a^	3.87 ± 0.6 ^a^	4.12 ± 0.6 ^a^
N7	3.62 ± 1.1 ^a^	3.37 ± 0.7 ^ab^	4.25 ± 0.7 ^a^	3.75 ± 0.5 ^a^
F7	3.75 ± 1.4 ^a^	3.50 ± 1.1 ^ab^	4.37 ± 0.7 ^a^	3.75 ± 0.9 ^a^

* Different small letters in each column indicate significant differences at the confidence level of 5%. ** N3: jelly containing 3% *w*/*w* nanoencapsulated CSE, F3: jelly containing 3% *w*/*w* free CSE, N7: jelly containing 7% *w*/*w* nanoencapsulated CSE, F7: jelly containing 7% *w*/*w* free CSE.

**Table 4:** Water solubility index and hygroscopicity of freeze-dried jelly powders containing CSE in free or nanocoated forms *.

Sample **	Water Solubility Index (%)	Hygroscopicity (g/100 g)
Control	41.12 ± 2.6 ^a^	9.36 ± 3.1 ^a^
N3	43.74 ± 2.1 ^a^	5.09 ± 1.6 ^b^
F3	42.52 ± 1.7 ^a^	3.41 ± 0.6 ^b^
N7	42.42 ± 1.7 ^a^	1.22 ± 0.6 ^b^
F7	39.59 ± 2.4 ^a^	1.52 ± 1.4 ^b^

* Similar small letters in each column indicate insignificant differences at the confidence level of 5%. ** N3: jelly containing 3% *w*/*w* nanoencapsulated CSE, F3: jelly containing 3% *w*/*w* free CSE, N7: jelly containing 7% *w*/*w* nanoencapsulated CSE, F7: jelly containing 7% *w*/*w* free CSE.

**Table 5:** Different jelly formulations containing *Capparis spinosa* fruit extract *.

Ingredients (% *w*/*w*)	Control	N3	F3	N7	F7
Gelatin	15	15	15	15	15
Sugar	75	75	75	75	75
Maltodextrin	7.5	4.5	4.5	1	1
Extract of *Capparis spinosa*	0	3	3	7	7
Fruit essence	2.5	2.5	2.5	2.5	2.5

* N3: jelly containing 3% *w*/*w* nanoencapsulated CSE, F3: jelly containing 3% *w*/*w* free CSE, N7: jelly containing 7% *w*/*w* nanoencapsulated CSE, F7: jelly containing 7% *w*/*w* free CSE.

## Data Availability

Data are contained within the article.
